# Factors associated with non-treatment of hypertension and gender differences at baseline in the ELSA-Brasil cohort

**DOI:** 10.1590/1414-431X2023e12937

**Published:** 2024-02-09

**Authors:** A.K.M. Néri, R.M.F. Xavier, S.M.A. Matos, M.C.C. Almeida, R.M. Ladeira, A.A. Lopes, D.O.C. Lino, A.P.P. Lázaro, R.V.B.M. Cairutas, J.H. Silva, J.M.O. Lima, M.C. Chaves, R.P. Silva, G.B. Silva

**Affiliations:** 1 Programa de Pós-Graduação em Saúde Coletiva, Centro de Ciências da Saúde, Universidade de Fortaleza, Fortaleza, CE, Brasil; 2 Serviço de Cardiologia, Hospital Universitário Walter Cantídio, Faculdade de Medicina, Universidade Federal do Ceará, Fortaleza, CE, Brasil; 3 Instituto de Saúde Coletiva, Universidade Federal da Bahia, Salvador, BA, Brasil; 4 Instituto Gonçalo Moniz, Fundação Oswaldo Cruz, Salvador, BA, Brasil; 5 Hospital João XXIII, Fundação Hospitalar do Estado de Minas Gerais, Secretaria Estadual de Saúde, Belo Horizonte, MG, Brasil; 6 Departamento de Medicina Interna/Nefrologia, Universidade Federal da Bahia, Salvador, BA, Brasil

**Keywords:** Hypertension, Treatment, Medication therapy management, Gender analysis in health, Gender equity

## Abstract

The treatment of arterial hypertension (AH) contributes to the reduction of morbidity and mortality. Gender differences are likely to play a role, as non-treatment is associated with clinical and sociodemographic aspects. The aim of this study was to investigate the factors associated with non-treatment of AH and gender differences in hypertensive individuals from the ELSA-Brasil cohort. The study was conducted with 5,743 baseline hypertensive cohort participants. AH was considered if there was a previous diagnosis or if systolic blood pressure (SBP) was ≥140 and/or diastolic BP (DBP) was ≥90 mmHg. Sociodemographic and anthropometric data, lifestyle, comorbidities, and use of antihypertensive medications were evaluated through interviews and in-person measurements. Treatment with renin-angiotensin-aldosterone system inhibitors (RAASi) or other antihypertensive medications and non-treatment were evaluated with multivariate logistic regression. Non-treatment was observed in 32.8% of hypertensive individuals. Of the 67.7% treated individuals, 41.1% received RAASi. Non-treatment was associated with alcohol consumption in women (OR=1.41; 95%CI: 1.15-1.73; P=0.001), lowest schooling level in men (OR=1.70; 95%CI: 1.32-2.19; P<0.001), and younger age groups in men and women (strongest association in males aged 35-44 years: OR=4.58, 95%CI: 3.17-6.6, P<0.001). Among those using RAASi, a higher proportion of white, older individuals, and with more comorbidities was observed. The high percentage of non-treatment, even in this civil servant population, indicated the need to improve the treatment cascade for AH. Public health policies should consider giving special attention to gender roles in groups at higher risk of non-treatment to reduce inequities related to AH in Brazil.

## Introduction

Arterial hypertension (AH) is an independent risk factor for cardiovascular disease (CVD) with a great impact on the health of the global population and is a major risk factor for mortality in the general population (1). With an overall prevalence of 24.1% in men and 20.1% in women (1,2), it is a very common disease. Brazil has also experienced an increase in the prevalence of AH, currently affecting 27.3% of women and 21.2% of men (3).

Adequate treatment of AH is essential to reduce disease-related morbidity and mortality and consists of lifestyle changes and the use of anti-hypertensive medications (4), such as renin-angiotensin-aldosterone system (RAAS) inhibitors (RAASi), angiotensin-converting enzyme inhibitors (ACEIs), and angiotensin receptor blockers (ARBs). These medications play an important role in the prevention of CVD and chronic kidney disease (CKD), which is why they are considered the first-line of treatment of hypertension for many patients (4,5). Despite the proven efficacy of these medications, AH treatment and control rates remain unsatisfactory in most countries (6,7).

There are important gender differences related to AH prevalence, treatment, and control (8). A study showed that women had higher awareness of hypertension and the use of antihypertensive medications compared to men. However, this has not translated into better blood pressure (BP) control in women, showing that further studies are needed to analyze the gender differences related to AH management (9).

There are few studies on antihypertensive treatment patterns in Brazil. Therefore, more accurate information from a large sample such as the cohort from the Longitudinal Study of Adult Health (*Estudo Longitudinal de Saúde do Adulto* - ELSA-Brasil) on this topic is necessary to guide health policies for AH management (8,10). Therefore, this study aimed to investigate the factors associated with non-treatment of arterial hypertension (AH) and gender differences among the hypertensive individuals at baseline in the ELSA-Brasil cohort.

## Material and Methods

### Study design

ELSA-Brasil is a prospective cohort study carried out in six large urban centers in Brazil (in the municipalities of Belo Horizonte, Porto Alegre, Rio de Janeiro, Salvador, São Paulo, and Vitória). The study started in 2008-2010, when the baseline data were obtained. The present study is a cross-sectional study, as it analyzes baseline data of the cohort.

### Study population

A total of 15,105 participants aged between 35 and 74 years were recruited from the cohort, which included active or retired civil servants from higher education institutions in the urban centers cited above. Those who intended to leave the institution, retirees living outside the metropolitan area of the research center, those with severe cognitive or communication deficits, and pregnant women or women who had a pregnancy less than 4 months before the initial interview were not included.

The study included only hypertensive individuals at baseline from the cohort. AH was considered for those who reported previous disease, except women diagnosed only during the pregnancy, those with a casual systolic BP (SBP) measurement ≥140 mmHg and/or casual diastolic BP (DBP) ≥90 mmHg, and/or those using antihypertensive medications in the previous two weeks (10).

**Figure 1:**
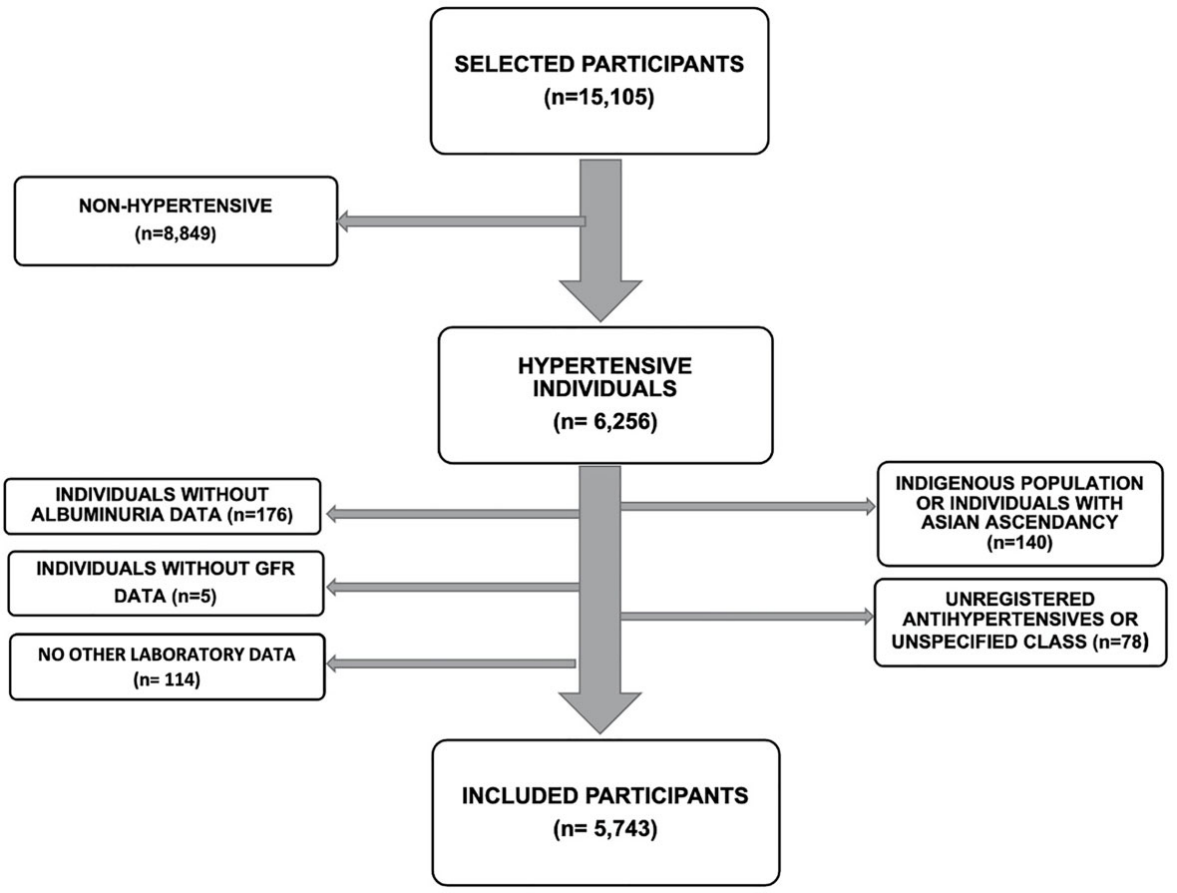
Criteria used for the study sample selection. GFR: glomerular filtration rate.

Those who did not have data from laboratory tests analyzed by us, those who did not have available renal function indicators (albumin/creatinine ratio - ACR and/or glomerular filtration rate - GFR), those who were using antihypertensive medications but whose class of medication used was not specified, and those who reported indigenous ethnicity or Asian descent (due to the small number of individuals of these ethnicities in the cohort) were excluded. Figure 1 summarizes the sample selection.

### Clinical assessment

Questionnaires were applied in-person by trained and certified interviewers, and information was obtained on age, gender, self-reported ethnicity/skin color, level of schooling, smoking status and alcohol consumption, AH presence and time of diagnosis, comorbidities, such as diabetes mellitus (DM) and chronic kidney disease (CKD), and a past medical history of heart failure (HF), myocardial infarction (MI), and stroke.

As for AH treatment, the participants answered a questionnaire about the continuous use of antihypertensive medications in the two previous weeks, stating which medications were being used. The reported antihypertensive medications were classified into categories according to their main pharmacological action: ACEIs, ARBs, diuretics, beta-blockers, calcium-channel blockers, vasodilators (direct action), and central and peripheral sympatholytic medications.

The participants' anthropometric data were evaluated after overnight fasting and with an empty bladder. Weight was measured using an electronic scale (Toledo¯, model 2096PP, Brazil), with a capacity of 200 kg and an accuracy of 50 g, with the participant barefoot and wearing a standard uniform. Height was assessed barefoot, with the head, buttocks, and heels touching the wall, and keeping their eyes on the horizontal plane using a stadiometer (Seca¯, Germany) with a 1-mm accuracy, attached to a smooth wall without a baseboard. Body mass index (BMI) was calculated as recommended, using weight and height measurements. Waist circumference (WC) was measured with the participant in the upright position, breathing normally, with feet together, top (shirt) pulled up and arms crossed in front of the chest with a non-stretchable measuring tape at the midpoint between the lower edge of the last rib and the anterior superior iliac crest.

A casual BP measurement was obtained from the left arm after a five-minute rest using a validated oscillometric tensiometer (Omron HEM 705CPINT, USA) with the participant seated in a quiet and temperature-controlled environment (20-24°C), as recommended by the current guideline recommendations (11). Three measurements were performed at one-minute intervals and casual BP was obtained through the average of the last two of the three measurements.

### Laboratory tests

The collection of biological samples was standardized in all research centers, with the analyses being carried out at the Universidade de São Paulo. The following parameters were analyzed in fasting blood samples: creatinine (Cr), using the Jaffé method without deproteinization; glycosylated hemoglobin (hbA1c), by high-performance liquid chromatography; and fasting glycemia (FG), total cholesterol (TC), high-density lipoprotein-cholesterol (HDL-c), low-density lipoprotein-cholesterol (LDL-c), and triglycerides (TG), using the enzymatic colorimetric method. GFR was estimated as previously recommended (12).

ACR was assessed in urine after a 12-h urine collection and the immunochemical assay was used to measure urinary albumin.

### Study groups

The individuals were grouped into: 1) Hypertensive participants reporting use of RAASi, ACEI, or ARBs, alone or in association with other antihypertensive medications; 2) Hypertensive participants reporting use of anti-hypertensive medications of any other class, alone or in combination; 3) Hypertensive individuals reporting no antihypertensive treatment.

### Covariates

The following covariates were evaluated: age, gender, skin color/ethnicity, level of schooling, smoking status, alcohol consumption including alcohol abuse, DM history and time of diagnosis of AH and DM, previous MI, HF or stroke, presence of metabolic syndrome (MS), BMI, WC, SBP, DBP, GFR, FG, hbA1c, serum TC, HDL-c, LDL-c, and TG, urinary ACR, and blood pressure control.

The self-reported ethnicity/skin color was classified as Black, White, and Brown. The level of schooling was defined as elementary school, high school, and higher education. Participants who reported having smoked at least 100 cigarettes during their lifetime and still smoked were considered smokers, whereas those who were not currently smoking were considered non-smokers. The current consumption of alcohol was assessed, and alcohol abuse was defined as a weekly consumption greater than 140 g of alcohol for women and 210 g for men (13).

DM was defined as pre-existing diabetes and/or FG ≥7 mmol/L and/or hbA1c ≥48 mmol/mol (14). As for the BMI, individuals were considered underweight with a BMI<18.5 kg/m^2^, normal weight with a BMI from 18.5 to <25 kg/m^2^, overweight with a BMI between 25 and 30 kg/m^2^, and obese with a BMI ≥30 kg/m^2^. MS was defined according to the Joint Interim Statement criteria (15), considering the WC limits stipulated for South American individuals (≥90 cm for men, ≥80 cm for women). GFR <60 mL/min/1.73 m^2^ or ACR ≥30 mg/g of Cr was considered as renal dysfunction (16).

BP was considered controlled if SBP <130 and DBP <80 mmHg in diabetics, in those with ACR ≥30 mg/g of Cr, and in those with GFR<60 mL/min/1.73 m^2^ and if SBP<140 and DBP<90 mmHg for all other participants who did not fit the characteristics described above (10,11).

### Statistical analysis

The statistical analyses were performed using the Statistical Package for Social Sciences (SPSS¯) for Windows, version 24.0 (SPSS Incorporation, IBM, USA). For descriptive statistics, continuous variables are reported as means±SD and quartiles. To assess clinical and sociodemographic factors associated with the non-treatment of AH, the Kruskal-Wallis test was used to test the difference in quantitative variables across AH treatment groups. Pearson's chi-squared test was used to verify the relationship of dependence between the variables, considering the standard treatment groups for AH. Adjusted odds ratios with 95% confidence intervals (95%CI) were obtained with the multivariate analysis using logistic regression.

### Ethical aspects

The ELSA-Brasil study was approved by the National Research Ethics Commission of the Brazilian National Health Council, in addition to the approval by the Ethics Committees of the six research centers (Federal University of Bahia, Federal University of Minas Gerais, Federal University of Porto Alegre, Federal University of Espírito Santo, University of São Paulo, and Oswaldo Cruz Foundation - FioCruz). The participants were informed about the purposes, stages, risks, and benefits of the study and, after such clarification, they signed the free and informed consent form before being enrolled in the study.

## Results

Of the 15,105 individuals in the cohort, 5,743 hypertensive people were included, most of them White (45.7%), with a higher education (50.2%), male (50.7%), with a mean age of 55.4 years (IQR 49-62) years and mean time of AH diagnosis of 10.5 years (IQR 3-15). As for AH treatment, 67.6% were undergoing treatment and only 37.3% of the participants had controlled BP. The use of RAASi was reported by 41.1 and 32.8% were untreated hypertensive participants.

We observed a higher percentage of non-treatment among younger individuals, those with lower BMI, smokers, and those with uncontrolled BP (all with P<0.001). We also observed a shorter time of AH diagnosis (4, IQR 2-10 years, P<0.001) and a higher GFR (85.3, IQR 74.9-96.2 mL/min/1.73m^2^, P<0.001) among those who were not treated. White individuals, those in older age groups, overweight and obese individuals, and those with more comorbidities such as HF, stroke, and MI showed a higher proportion of ACEIs/ARBs treatment (all with P<0.001).

We observed a higher proportion of non-treatment among Black and Brown men, those with high school education, smokers, those in younger age groups, with lower BMI, and shorter time of AH (all with P<0.001), and among alcohol abusers (P=0.039). In women, there was a higher proportion of non-treatment among those of Black ethnicity and those with lower WC, TG, and fasting blood glucose levels (all with P<0.001). Among men and women with blood pressure control, a lower proportion of untreated individuals was observed (P<0.001 for both). In the female group, a predominance of RAASi use was also observed with all levels of schooling (P=0.044), in all age groups, with the exception of those aged 35-44 years (P<0.001). All these data are reported in Supplementary Table S1.

**Figure 2:**
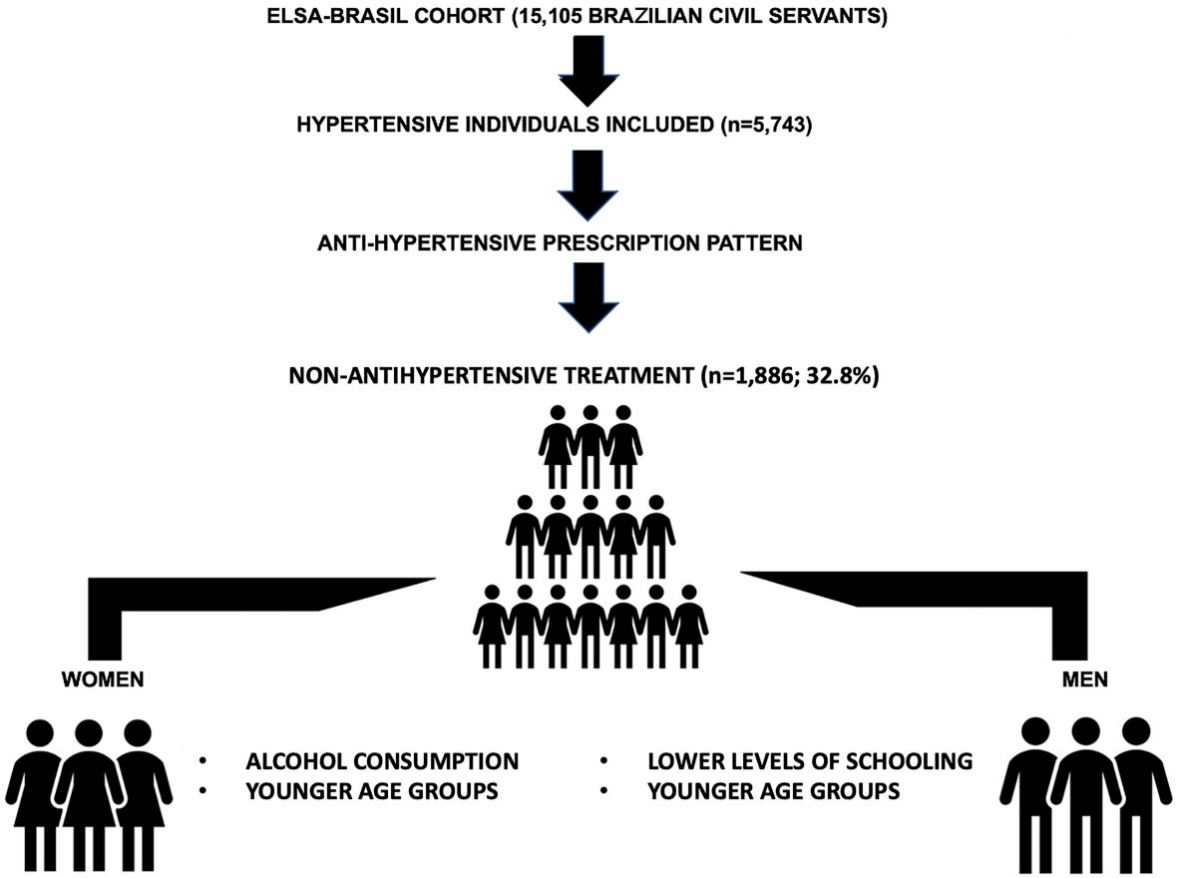
Main results of the study comparing hypertensive men and women from the ELSA-Brasil cohort.

Factors independently associated with non-treatment were younger age (mainly the age group of 35-44 years: OR=3.75, 95%CI: 2.86-4.92, P<0.001), lower levels of schooling (mainly elementary level: OR=1.51, 95%CI: 1.24-1.85, P<0.001), overweight (OR=1.34, 95%CI: 1.15-1.57, P<0.01), and alcohol consumption (OR=1.29, 95%CI: 1.12-1.5, P<0.001). Among men, lower levels of schooling (elementary: OR=1.70, 95%CI: 1.32-2.19, P<0.001 and high school: OR=1.60, 95% CI: 1.3-1.96, P<0.001) and younger age groups (35 to 44 years: OR=4.58, 95%CI: 3.17-6.6, P<0.001 and 45 to 54 years: OR=2.5, 95%CI: 1.85-3.37, P<0.001), and among women, alcohol consumption (OR=1.41, 95%CI: 1.15-1.73, P=0.001) and younger age groups (35 to 44 years: OR=2.88, 95%CI: 1.94-4.27, P<0.001 and 45 to 54 years: OR=1.61, 95%CI: 1.18-2.21, P=0.003) were independently associated with non-treatment (Table 1). The main results of the study are shown in Figure 2.

**Table 1:** Factors independently associated with non-treatment of hypertension among baseline hypertensive individuals from the ELSA-Brasil cohort, 2008-2010.

Variables	Overall population			Men			Women		
	OR	95%CI	P-value	OR	95%CI	P-value	OR	95% CI	P-value
Age groups (years)									
35-44	3.75	2.86-4.92	<0.001	4.58	3.17-6.6	<0.001	2.88	1.94-4.27	<0.001
55-64	1.36	1.09-1.7	0.006	1.49	1.1-2.01	0.010	1.25	0.91-1.71	0.166
Level of schooling									
Elementary	1.51	1.24-1.85	<0.001	1.70	1.32-2.19	<0.001	-	-	-
High School	1.28	1.1-1.49	0.001	1.60	1.3-1.96	<0.001	-	-	-
Body mass index categories									
<18.5 kg/m^2^	1.32	1.08-1.61	0.007	1.55	1.16-2,08	0.003	-	-	-
18.5-24.9 kg/m^2^	1.34	1.15-1.57	<0.001	1.52	1.22-1.88	0.000	-	-	-
Alcohol consumption	1.29	1.12-1.5	0.001	-	-	-	1.41	1.15-1.73	0.001
Absence of metabolic syndrome	2.87	2.4-3.45	<0.001	2.64	2.02-3.46	<0.001	3.20	2.54-4.04	<0.001
Non-controlled arterial pressure	3.10	2.67-3.6	<0.001	3.59	2.89-4.46	<0.001	2.54	2.06-3.14	<0.001
Absence of heart failure	2.47	1.42-4.29	0.001	3.06	1.48-6.34	0.003	-	-	-
Absence of diabetes mellitus	1.72	1.45-2.05	<0.001	1.76	1.4-2.23	<0.001	1.71	1.32-2.22	<0.001
Absence of myocardial infarction	2.83	1.71-4.7	<0.001	2.97	1.57-5.62	0.001	2.78	1.3-5.94	0.008
Glomerular filtration rate ≥60 mL/min/1.73 m^2^	1.78	1.34-2.35	<0.001	2.03	1.39-2.96	<0.001	-	-	-

Adjusted odds ratios (OR) with 95% confidence intervals (95%CI) were obtained with the multivariate analysis using logistic regression.

## Discussion

This study included one of the largest sample sizes on hypertension treatment carried out in Brazil. It was demonstrated that a third of hypertensive individuals were not being treated for AH and that sociodemographic factors were independently associated with non-treatment. Important gender differences were observed, since among men, a lower level of schooling and, among women, the use of alcohol were independently associated with non-treatment. In men and women, younger age was independently associated with non-treatment, with a stronger association in males. The most frequently used antihypertensive medications were the RAASi and there was a greater proportion of white, older people, and participants with more comorbidities using these medications.

The pattern of antihypertensive medication prescription varies greatly between studies. A Chinese study showed that calcium channel blockers were the most prescribed medications in the evaluated population (17). Other studies showed that ACEi/ARBs were the most often prescribed medications among young or elderly adults (18,19). In our study, there was a greater proportion of RAASi use among the participants from the older age groups, of White ethnicity, and those with comorbidities. This pattern must have occurred because the findings disclosed by large previous studies have already demonstrated greater renal and cardiovascular protection in patients with this profile who were using RAASi (4-6).

Despite the known benefits of ACEi/ARB in improving cardiovascular and renal outcomes, it is important to remark that it has also been demonstrated that renin cells can lead to renal arteriolar hypertrophy that is indistinguishable from that observed in uncontrolled hypertension when these cells are chronically stimulated by RAAS gene mutations or by the use of RAASi in humans and other mammals. This kidney injury is associated with lower GFR, lower renal plasma flow, kidney fibrosis, up-regulation of sodium transporters, impaired sodium excretion, and salt-sensitive hypertension. These findings suggest that it would be important to conduct prospective randomized controlled studies with histological evaluation to determine the extent of renal damage caused by the widespread use of RAASi (20,21).

It is believed that the significant percentage of non-treatment found in this study comprised participants who were previously diagnosed with AH and did not adhere to treatment or who were undiagnosed hypertensive individuals. Studies have shown even higher percentages of non-treatment of hypertension than those demonstrated in the present study, such as the study by Rauniyar et al. (22), which found 73.6 and 79.8% of non-treatment among hypertensive individuals in India and Nepal, respectively. The same study showed a large percentage of unawareness of AH (69.7% in India and 60% in Nepal). It also identified that only 17.8% (in India) and 10.4% (in Nepal) of the individuals undergoing treatment had their BP controlled.

Unlike our study, an investigation that used telephone survey data in capital cities of Brazil showed that the prevalence of antihypertensive medication use was higher than that demonstrated by us in the assessed years (79.6% in 2011, 79% in 2014, and 80% in 2017) (23). This divergence may have occurred due to the temporal difference between our sample evaluated (2008 to 2010) and the one assessed in the study by Leitão et al. (24) (2011 to 2017), since it is believed that there was an increase in care of hypertensive patients due to the expansion of the family health programs and the consequent improvement in primary care in Brazil over the years (24). Nevertheless, a higher percentage of treated hypertensive participants was expected in our sample, given the fact that it comprises Brazilian civil servants, who generally have more access to information and health services (10).

In this study, younger age, a lower level of schooling, overweight, and current alcohol use were independently associated with non-treatment. A study with young hypertensive individuals showed that 11.3% of men and 26.2% of women were receiving treatment for hypertension and that it was controlled in 7.4% of men and 18.4% of women. This shows the high rate of untreated hypertensive patients, especially among young people (25). Additionally, the findings of our study may be related to the fact that older age and the presence of comorbidities are associated with greater demand for health services in Brazil (26). Younger individuals, therefore, seek health services less often and may not be aware of their health status, as they may have the belief that they are less likely to suffer from chronic non-communicable diseases (NCDs) (27).

Young people's low demand for health services is of particular importance for young hypertensive women of childbearing age, since pre-gestational counseling and adequate follow-up of this group during pregnancy is essential for adequate control of AH and the consequent reduction in maternal and fetal morbidity and mortality. The most adequate medication treatment for AH in this population should always be implemented even in the pre-pregnancy counseling phase, as some medications, such as RAASi, carry a risk of teratogenicity to the conceptus and should, therefore, be avoided (5,28).

It is known that alcohol consumption is associated with lower disease awareness, lower treatment adherence, and therefore, non-treatment of AH (29-32). A study evaluated the association between alcohol abuse and increased risk of treatment abandonment in chronic NCDs and showed that there was an association between alcohol abuse and non-treatment of hypertension and a gradual and linear decrease in adherence to these medications with increasing levels of alcohol use (33). Although the present study did not demonstrate an association between alcohol abuse and non-treatment, there was an association between the latter and current alcohol consumption, which could be justified, among other causes, by a possible non-adherence to treatment among the individuals who consumed alcohol.

It has been shown that women have a lower risk of developing high-risk drinking patterns than men (34); however, it is known that women are more vulnerable to the effects of alcohol than men and they are more likely to develop dependence (35). Contrary to what was shown by Kim et al. (25), although we did not observe an association between non-treatment and excessive alcohol consumption in women in our research, we detected an independent association between current alcohol use and non-treatment for AH in the female population. It is possible that the association between alcohol consumption and lower disease awareness and treatment adherence and a greater chance of non-treatment for AH reported above (29-32) may be maximized in women, given their greater vulnerability to the effects of alcohol consumption (35).

A study on the potential effects of a program to improve AH detection and screening for alcohol abuse to promote better AH treatment estimated that if 50% of men who are unaware of their AH were informed about their condition and received the standard treatment and if 50% of these men with AH were treated for alcohol abuse, the percentage of uncontrolled AH would be reduced by 8.6% in men, with alcohol use intervention accounting for approximately 33% of this improvement. In women, the percentage of uncontrolled AH would decrease 7.4%, but the effect attributable to the treatment for alcohol abuse would be greater (40%) compared to men (36). This study suggests that an intervention for better screening and approach to AH and alcohol consumption among women, a group that would in theory be more vulnerable to alcohol addiction, could lead to good results and reinforces the need to address this issue.

A study found a positive association between AH and level of schooling, in addition to a lower percentage of treated hypertensive men with a low level of schooling (37). Another study revealed that, among those with a lower level of schooling, there was a higher percentage of AH treatment among women and that among men, the use of antihypertensive medications was positively correlated with higher level of schooling (9). Another research showed that having high-school and elementary school increased the chances of being treated for AH among Chinese men (38). Among the men in the present study, a lower level of schooling was independently associated with non-treatment for AH, revealing that it is still necessary to carry out more in-depth investigations related to this topic.

It is known that inequalities in the management of CVD and AH decrease as a country becomes wealthier; however, there is considerable variation in patterns of inequality related to wealth, even between countries with similar levels of economic development, indicating a fundamental role of health systems in improving the adequate management of AH. It is supposed, therefore, that a more equitable control of AH can be achieved even in countries with limited resources such as Brazil with the use of health policies adapted to national contexts, aiming to achieve the ideal impact at the population level (39,40).

In conclusion, the high percentage of non-treatment observed in a population of civil servants indicated the need to improve the cascade of care for hypertensive individuals. Public health policies should pay special attention to gender roles among groups at higher risk of non-treatment, aiming at reducing inequities related to AH approach in Brazil.

### Study limitations

This study has limitations and the main one is related to its cross-sectional design. Although the ELSA-Brasil is a cohort study, only baseline data were analyzed in the present study. In addition, factors that could justify some of the gender differences such as the use of health services and treatment adherence were not assessed in the present study.

However, our findings are relevant as they address the treatment profile of hypertensive individuals and particularly the aspects related to non-treatment and gender differences in the largest and most relevant Brazilian cohort, whose main objects of study are chronic NCDs. These data are important because they can improve our knowledge on the subject and can be used as the basis to modify Brazilian public health policies regarding the management of AH, which, in the future, could actively identify individuals at greater risk of developing target-organ damage or adverse cardiovascular events resulting from untreated AH.

## Supplementary Material

Click to view [pdf].
