# Supplementary material for: Factors associated with non-treatment of hypertension and gender differences at baseline in the ELSA-Brasil cohort
Source: Braz J Med Biol Res. 2024 Feb 9;57:e12937. doi: 10.1590/1414-431X2023e12937 (PMC10868185; doi:10.1590/1414-431X2023e12937)
Supplement: Supplementary file 1 [file 1414-431X-bjmbr-57-e12937-suppl1.pdf]

Supplementary Table S1. Sociodemographic and clinical characteristics of men and women according to treatment groups regarding baseline hypertensive individuals from the ELSA-Brasil cohort, 2008–2010.

Variables	Total (n=5743)	Men				Women			
		Untreated (n=1102)	Treated with RAASi (n=1254)	Treated with other medications (n=558)	P-value	Untreated (n=784)	Treated with RAASi (n=1106)	Treated with other medications (n=939)	P-value
**Sociodemographic**									
Age range (years)*					<0.001				<0.001
35–44	647	216 (59.8)	101 (28)	44 (12.2)		134 (46.9)	79 (27.6)	73 (25.5)	
45–54	2062	489 (45.2)	417 (38.5)	176 (16.3)		307 (31.3)	356 (36.3)	317 (32.3)	
55–64	2047	297 (30.7)	464 (47.9)	207 (21.4)		256 (23.7)	452 (41.9)	371 (34.4)	
65–74	987	100 (19.9)	272 (54.1)	131 (26)		87 (18)	219 (45.2)	178 (36.8)	
Ethnicity/skin color*					<0.001				0.530
Black	1540	259 (42)	232 (37.7)	125 (20.3)		240 (26)	359 (38.9)	325 (35.2)	
Brown	1519	343 (41)	337 (40.3)	157 (18.8)		198 (29)	266 (39)	218 (32)	
White	2583	478 (34)	659 (46.9)	267 (19)		330 (28)	467 (39.6)	382 (32.4)	
Level of schooling*					<0.001				0.044
Elementary	847	208 (42.1)	208 (42.1)	78 (15.8)		83 (23.5)	153 (43.3)	117 (33.1)	
High school	2015	430 (44.5)	357 (36.9)	180 (18.6)		282 (26.9)	431 (41.1)	335 (32)	
Higher education	2881	464 (31.9)	689 (47.4)	300 (20.6)		419 (29.3)	522 (36.6)	487 (34.1)	
**Behavioral**									
Smoking*	729	207 (50.5)	133 (32.4)	70 (17.1)	<0.001	103 (32.3)	126 (39.5)	90 (28.2)	0.067
Alcohol consumption*	3808	857 (38.9)	944 (42.9)	400 (18.2)	0.028	496 (30.9)	581 (36.2)	530 (33.0)	<0.001
Alcohol abuse*	545	184 (42.8)	178 (41.4)	68 (15.8)	0.039	37 (32.2)	47 (40.9)	31 (27)	0.309
**Anthropometric**									
BMI category (kg/m^2)*					<0.001				<0.001
<18.5	16	10 (83.3)	2 (16.7)	0 (0)		0 (0)	1 (25.0)	3 (75.0)	
18.5–24.9	1296	291 (45)	231 (35.8)	124 (19.2)		222 (34.2)	230 (35.4)	198 (30.5)	
25.0–29.9	2447	512 (38)	579 (42.9)	258 (19.1)		296 (27.0)	417 (38.0)	385 (35.1)	
≥30	1982	289 (31.9)	441 (48.7)	176 (19.4)		266 (24.7)	458 (42.6)	352 (32.7)	
Waist circumference (cm)[#]	95.7 (87.6–104.5)	96 (89.1–103.7)[a]	100.7 (93.4–108.2)[b]	99.5 (92.5–107.4)[b]	<0.001	89.9 (82–99)[a]	93 (85.5–102.4)[b]	92.5 (84.5–101.4)[b]	<0.001
**Comorbidities**									
Heart failure*	183	11 (11.3)	57 (58.8)	29 (29.9)	<0.001	10 (11.6)	36 (41.9)	40 (46.5)	0.001
Stroke*	139	10 (14.5)	44 (63.8)	15 (21.7)	<0.001	12 (17.1)	39 (55.7)	19 (27.1)	0.013
Myocardial infarction*	194	14 (12.7)	66 (60)	30 (27.3)	<0.001	8 (9.5)	38 (45.2)	38 (45.2)	0.001
Diabetes mellitus*	1860	296 (29.3)	507 (50.1)	208 (20.6)	<0.001	151 (17.8)	389 (45.8)	309 (36.4)	<0.001
GFR <60 mL/min/1.73m^2*	482	49 (18.8)	145 (55.8)	66 (25.4)	<0.001	35 (15.8)	99 (44.6)	88 (39.6)	<0.001
ACR ≥30 mg/g of Cr*	493	94 (32.2)	145 (49.7)	53 (18.2)	0.045	55 (27.4)	90 (44.8)	56 (27.9)	0.161
Metabolic syndrome*	4398	770 (33.2)	1079 (46.5)	470 (20.3)	<0.001	445 (21.4)	883 (42.5)	751 (36.1)	<0.001

**Clinical-laboratory**									
Controlled blood pressure*	2147	180 (20.5)	482 (54.8)	217 (24.7)	<0.001	244 (19.2)	529 (41.7)	495 (39)	<0.001
Time of AH diagnosis#	8 (3–15)	4 (2–10)[a]	9 (4–15)[b]	10 (4–17)[b]	<0.001	5 (2–11)[a]	9 (4–17)[b]	10 (5–18)[b]	<0.001
SBP (mmHg)#	131 (120–143.5)	140 (130–149.5)[a]	129.5 (119–142.5)[b]	130.8 (120.5–142.5)[b]	<0.001	133 (121.5–144.5)[a]	126.8 (115.5–139.5)[b]	125 (115.5–138)[b]	<0.001
DBP (mmHg)#	82 (74.5–90)	90 (82–95)[a]	81 (74.5–89)[b]	81 (73.5–88.5)[b]	<0.001	84.5 (77–91.5)[a]	78.5 (72–85.5)[b]	78 (71.5–85)[b]	<0.001
Total cholesterol (mmol/L)#	5.51 (4.81–6.26)	5.64 (4.96–6.44)[a]	5.25 (4.60–6.02)[b]	5.33 (4.60–6.05)[b]	<0.001	5.64 (4.91–6.44)	5.56 (4.89–6.28)	5.59 (4.89–6.28)	0.102
HDL-c (mmol/L)#	1.34 (1.16–1.60)	1.29 (1.11–1.47)[a]	1.22 (1.06–1.40)[b]	1.24 (1.06–1.42)[b]	<0.001	1.53 (1.32–1.81)[a]	1.47 (1.27–1.73)[b]	1.45 (1.27–1.71)[b]	<0.001
LDL-c (mmol/L)#	3.31 (2.74–3.96)	3.52 (2.90–4.11)[a]	3.18 (2.61–3.786)[b]	3.16 (2.64–3.75)[b]	<0.001	3.44 (2.82–4.09)	3.34 (2.77–3.98)	3.34 (2.74–3.96)	0.096
Triglycerides (mmol/L)#	1.48 (1.06–2.11)	1.65 (1.15–2.36)	1.66 (1.19–2.37)	1.60 (1.16–2.33)	0.747	1.24 (0.90–1.79)[a]	1.38 (1.02–1.87)[b]	1.38 (0.99–1.88)[b]	<0.001
Fasting glycemia (mmol/L)#	6.1 (5.66–6.71)	6.1 (5.66–6.66)[a]	6.32 (5.82–7.21)[b]	6.27 (5.82–6.99)[b]	<0.001	5.77 (5.38–6.27)[a]	5.99 (5.55–6.66)[b]	6.05 (5.55–6.66)[b]	<0.001
hbA1C (mmol/mol)#	37 (32–42)	5.4 (30–40)[a]	37 (32–44)[b]	37 (32–42)[b]	<0.001	36 (31–41)[a]	5.6 (32–43)[b]	37 (32–42)[b]	<0.001
GFR (mL/min/1.73m^2)#	82.1 (71–93)	84.4 (74.5–94.4)[a]	78.4 (68.1–89.9)[b]	78.9 (68.5–90.5)[b]	<0.001	86.5 (74.9–98.3)[a]	82.8 (71.8–92.8)[b]	80.7 (69.4–91.6)[b]	<0.001
ACR (mg/g of Cr)#	6.7 (5–9.4)	5.1 (4.3–7.2)	5.2 (4.3–7.7)	5.4 (4.4–7.6)	0.066	7.8 (6.4–9.7)	7 (6.6–10.3)	7.9 (6.6–9.7)	0.169

RAASi: Renin-angiotensin-aldosterone system inhibitors; BMI: body mass index; GFR: glomerular filtration rate; AH: arterial hypertension; SBP: systolic blood pressure; DBP: diastolic blood pressure; HDL-c: high-density lipoprotein-cholesterol; LDL-c: low-density lipoprotein-cholesterol; HbA1C: glycosylated hemoglobin A1c; ACR: albumin/creatinine ratio. Categorical variables are reported as n (%); numerical variables reported as median and interquartile range. Different superscript letters indicate statistically different measures. *Chi-squared test. #Kruskal-Wallis test.
